# Supplementary figures and images for: EEG Signatures of Auditory Distraction: Neural Responses to Spectral Novelty in Real-World Soundscapes
Source: eNeuro. 2025 Jul 17;12(7):ENEURO.0154-25.2025. doi: 10.1523/ENEURO.0154-25.2025 (PMC12301874; doi:10.1523/ENEURO.0154-25.2025)

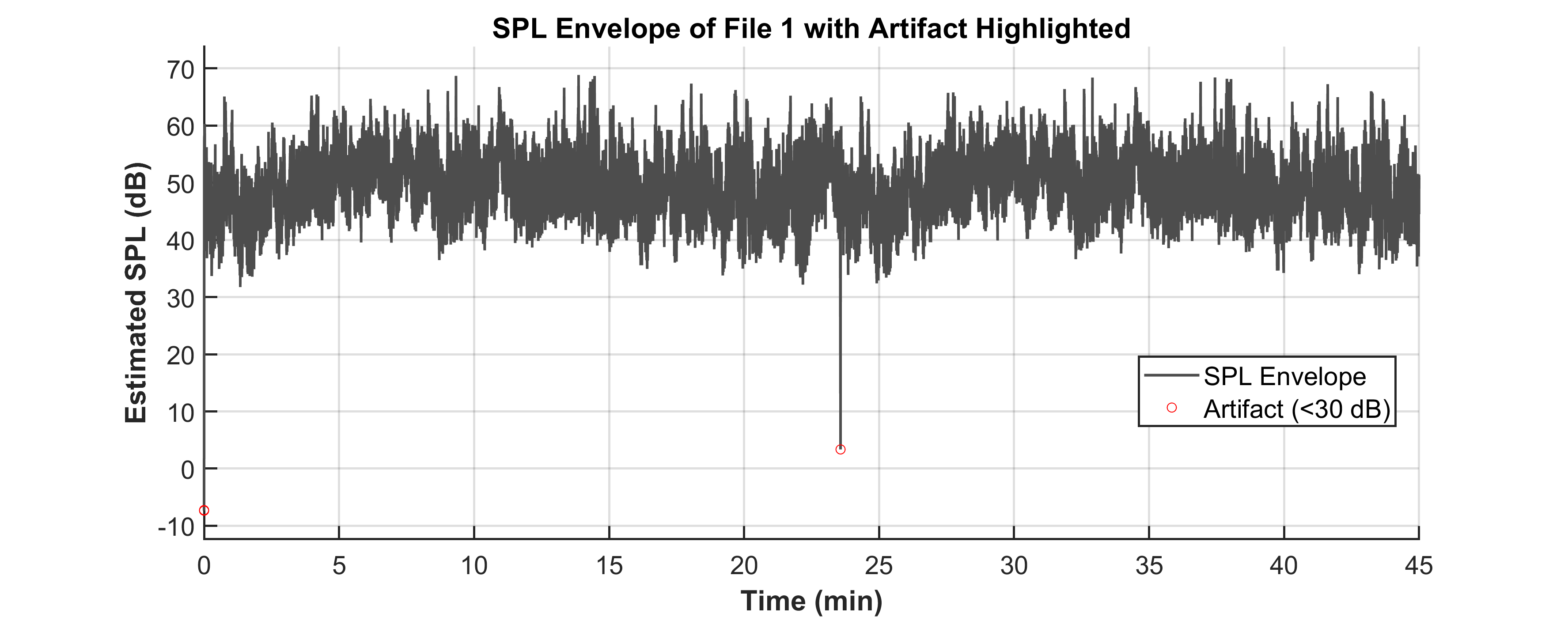

Supplement: Figure 1-1 — Sound level envelope of File 1 with artifact values included. Two brief dips below 30 dB(A) (highlighted in red) were identified as likely signal artifacts rather than valid acoustic events and were excluded from the cleaned analysis. These artifacts occurred only in File 1 and are shown here for transparency. Download Figure 1-1, TIF file. [file eneuro-12-ENEURO.0154-25.2025-s003.tif]

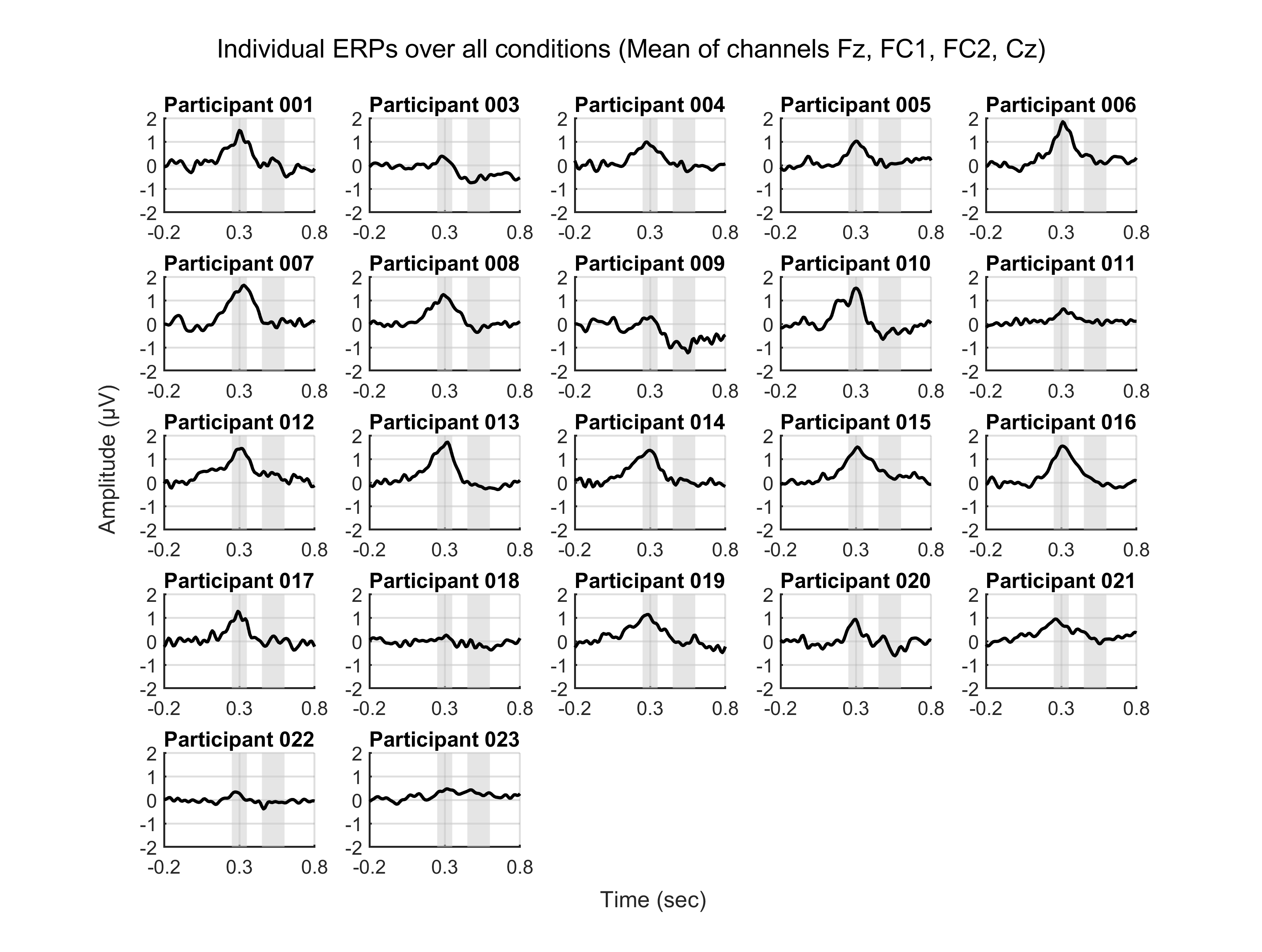

Supplement: Figure 6-1 — Individual ERPs for each participant, averaged across all conditions at selected frontocentral electrodes (Fz, FC1, FC2, and Cz). Shaded regions indicate the time windows of interest: P3a (250–350 ms, light gray) and Reorienting Negativity (RON, 450–600 ms, dark gray). Download Figure 6-1, TIF file. [file eneuro-12-ENEURO.0154-25.2025-s004.tif]

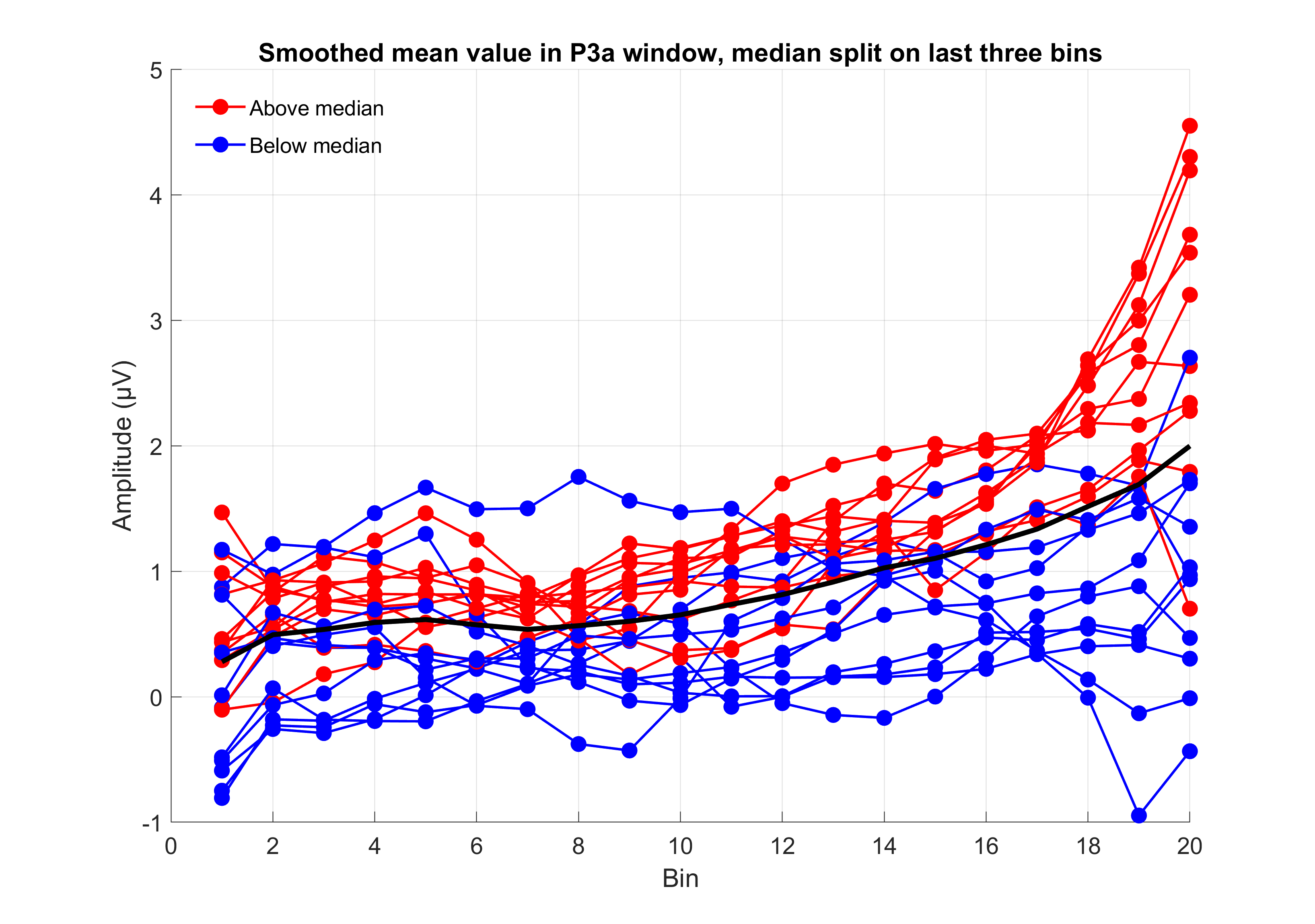

Supplement: Figure 7-1 — Smoothed mean EEG amplitude in the P3a window across spectral novelty bins and averaged over all conditions, split by median amplitude in the highest novelty bins. Red lines represent participants with amplitudes above the median, blue lines represent participants below the median, and the black line shows the grand average. This figure illustrates the overall trend of increasing EEG amplitude with spectral novelty, with inter-individual variability in the magnitude of this effect. Download Figure 7-1, TIF file. [file eneuro-12-ENEURO.0154-25.2025-s005.tif]
